# Influence of Laser Power and Rotational Speed on the Surface Characteristics of Rotational Line Spot Nanosecond Laser Ablation of TC4 Titanium Alloy

**DOI:** 10.3390/ma17174271

**Published:** 2024-08-29

**Authors:** Shunquan Shen, Xiaoxiao Chen, Jianbo Chen, Wenwu Zhang

**Affiliations:** 1 Faculty of Electrical Engineering and Computer Science, Ningbo University, Ningbo 315021, China; shenshunquan@nimte.ac.cn; 2 Ningbo Institute of Materials Technology and Engineering, Chinese Academy of Sciences, Ningbo 315201, China; chenjianbo@nimte.ac.cn (J.C.); zhangwenwu@nimte.ac.cn (W.Z.); 3 University of Chinese Academy of Sciences, Beijing 100049, China

**Keywords:** TC4 titanium alloy, laser ablation characteristics, rotating laser processing, laser line spot

## Abstract

The TC4 titanium alloy is widely used in medical, aerospace, automotive, shipbuilding, and other fields due to its excellent comprehensive properties. As an advanced processing technology, laser processing can be used to improve the surface quality of TC4 titanium alloy. In the present research, a new type of rotational laser processing method was adopted, by using a beam shaper to modulate the Gaussian spot into a line spot, with uniform energy distribution. The effects of the laser power and rotational speed on the laser ablation surface of the TC4 titanium alloy were analyzed. The results reveal that the melting mechanism of the material surface gradually changes from surface over melt to surface shallow melt with the increase in the measurement radius and the surface roughness increases first, then decreases and, finally, tends to be stable. By changing the laser power, the surface roughness changes significantly with the variation in the measurement radius. Because low laser power cannot provide sufficient laser energy, the measurement radius corresponding to the surface roughness peak of the microcrack area is reduced. Under a laser power of 11 W, the surface roughness reaches its peak when the measurement radius is 600 μm, which is 200 μm lower than that of a laser power of 12 W, 13 W, and 14 W. By changing the rotational speed, the centrifugal force generated by the rotation of the specimen affects the distribution and re-condensation of the molten pool of the surface. As the rotational speed increases, the shallow pit around the pit is made shallower by the filling of the pit with molten material and the height of the bulge decreases, until it disappears. The surface oxygen content of the material increases first and then decreases with the increase in the measurement radius and gradually approaches the initial surface state. Compared with a traditional laser processing spot, the rotational line spot covers a larger processing area of 22.05 mm^2^. This work can be used as the research basis for rotational modulation laser polishing and has significance for guiding the innovative development of high-quality and high-efficiency laser processing technology.

## 1. Introduction

Titanium alloy is an important structural metal with high strength, corrosion resistance, and high-temperature resistance. It has been widely used in aerospace, machinery manufacturing, automotive, shipping, the chemical industry, medical equipment, and other fields [1,2]. The usage of TC4 titanium alloy accounts for 75~85% of all titanium alloys. The TC4 titanium alloy is widely used due to its excellent comprehensive properties, such as in the creation of aerospace aircraft turbine blades and rocket engine components in the aerospace field [3], or auxiliary devices and artificial bones in the biomedical field [4]. Many studies have shown that the surface roughness of alloy components will affect their performance in service. Wang et al. [5] analyzed the influence of surface roughness on the fatigue life by changing the surface roughness of the TC17 titanium alloy impeller, by using a machine learning algorithm and finite element simulation analysis. The results show that when the surface roughness is large, the stress concentration area increases, dislocations and displacements occur at grain boundaries, and cracks and fractures easily occur. Liu et al. [6] carried out an erosion wear experiment on aluminum alloy and analyzed the influence of different surface roughness on the erosion wear of a compressor main blade and splitter blade. The results show that with the increase in surface roughness, the wear concentration area of the main blade and splitter blade diffuses from 80% of the blade height to 30% and 50%, respectively. The maximum wear rate of the main blade and splitter blade increases with the increase in the surface roughness. In the medical field, controlling the surface roughness can make the adhesion of cells on the surface of titanium alloy implants better [4]. Therefore, controlling the surface roughness of difficult-to-machine metal surfaces is one of the key issues to improve the performance of components in service.

The traditional methods to reduce the surface roughness of titanium alloy are mechanical polishing, chemical polishing, and electrochemical polishing [7,8,9,10,11,12]. Among them, due to the low processing efficiency of mechanical polishing, the polishing time and intensity are difficult to control, so scratches or cracks inevitably occur on the surface of the material; chemical polishing can deal with specimens with a complex surface structure, but the surface quality of the material after polishing is not good, and during the process of chemical polishing, it is easy to produce chemical pollution and cause damage to the environment. The pretreatment for electrochemical polishing is more complex, and the electrolyte used during the process presents problems, such as poor versatility, a short service life, strong corrosion, and difficult treatment. Laser polishing is a new type of polishing technology. It has the characteristics of high machining accuracy, non-contact, and no tool wear [13,14]. It performs selective material removal and material redistribution on the surface of the material through thermal and photochemical effects [15], and a smooth material surface can be obtained under the influence of surface tension and gravity [16].

In recent years, researchers have conducted some research on laser polishing of difficult-to-machine metal surfaces. Xu et al. [17] established a two-dimensional numerical model that considered heat transfer, fluid flow, material gasification, and volume expansion. The results show that the formation of a surface structure with 304 stainless steel is the result of the combined action of surface tension, recoil pressure, material removal, and volume expansion of the molten pool. Li et al. [18] used a nanosecond laser to polish the TA15 titanium alloy and found that the depth of and defects in the oxide layer produced by nanosecond pulse laser polishing are closely related to the laser power. Jaritngam et al. [19] used a nanosecond laser to polish the surface of the Ti6Al4V titanium alloy and studied the influence of different process parameters on the characteristics of the polished surface and subsurface. The experimental results show that the polishing effect can be greatly improved under the condition of low laser power and high laser repetition frequency. When a lower heat is applied to the surface of the material, the oxygen content of the recast layer will be reduced; the surface roughness of the initial surface affects the polishing effect during the test. Some progress has also been made in the research on new laser polishing technologies for titanium alloy. Wu et al. [20] proposed a new laser–chemical polishing method, which uses a nanosecond laser to polish the surface of the TC4 titanium alloy immersed in a phosphoric acid solution. Changing the number of laser scans can effectively control the hot corrosion and chemical corrosion rate of the material surface, greatly reducing the adverse effects of the recast layer and heat-affected zone in traditional laser polishing and achieving the selective removal of materials. The experimental results show that when the number of scans is 300, the surface roughness of the material decreases from 304 nm to 59 nm and the reduction rate is 81%. Kang et al. [21] used ultrasonic vibration-assisted laser polishing (UVLP) to polish 304 stainless steel and the experimental results were compared with those of traditional laser polishing (TLP). The experimental results show that under the same conditions as the traditional laser polishing process, UVLP reduces the surface roughness of 304 stainless steel from 2.777 μm to 0.551 μm, while TLP reduces the roughness to 0.703 μm. Compared with the traditional laser polishing process, the research on new technologies for hybrid polishing has made innovative breakthroughs, but it still cannot fully solve the contradiction between high efficiency and high quality.

Some research in the field of laser processing, using the method of beam modulation, have been carried out [22]. Laser beam shaping technology is widely used in laser additive manufacturing processes for metal. By adjusting the distribution of laser beam energy, the efficiency and quality of additive manufacturing can be improved [23]. Li et al. [24] used a spatial light modulator (SLM) to shape the geometry and intensity of a picosecond laser beam and verified the results for the modulated light by ablating the Ti6Al4V substrate. Yi et al. [25] used a beam shaper (DOE) to shape a nanosecond laser into a line spot and applied it to the laser polishing of nickel-based superalloys. The results show that the surface roughness of the material can be effectively reduced by polishing the surface of the nickel-based superalloy with a line spot and changing the number of scans.

In summary, laser polishing technology has broad application prospects and some breakthroughs have been made in the exploration of new laser polishing technology. However, laser polishing technologies with high efficiency and high integrity are rarely reported and the research on laser polishing technology based on beam modulation is very limited. In order to solve the contradiction between high-quality processing and high-efficiency processing and make a breakthrough in terms of a high-efficiency and high-quality laser polishing technology for material surfaces, the use of a laser spot after beam modulation was used to carry out rotational processing experiments, which cover a larger processing area of 22.05 mm^2^, and the rotational large-area ablation mechanism of the TC4 titanium alloy surface under different laser powers (11 W to 14 W) and rotational speed (0 RPM to 3000 RPM) conditions were analyzed.

## 2. Experimental Setting

**Figure 1:**
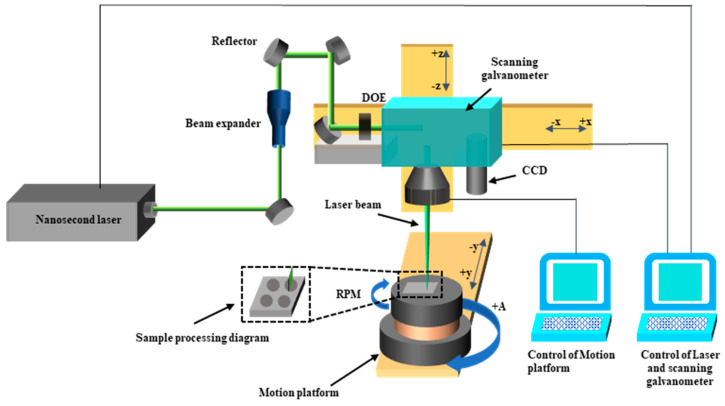
TC4 titanium alloy rotational nanosecond laser processing system and processing diagram.

The schematic diagram of the laser processing system is shown in Figure 1. The whole system consisted of a nanosecond pulse laser (PR-532-20B, Power Laser, Suzhou, China), a machine tool control system, a laser, and a scanning galvanometer control system. The pulses emitted by the nanosecond pulse laser, with a wavelength of 532 nm, pass through the beam shaper in the optical path. The beam shaper (DOE25-E-220511-SP, LBTEK, Changsha, China) converts the Gaussian beam through the microlens array into a line spot, with uniform energy distribution and a spot size of 14 μm × 5.3 mm. 

**Figure 2:**
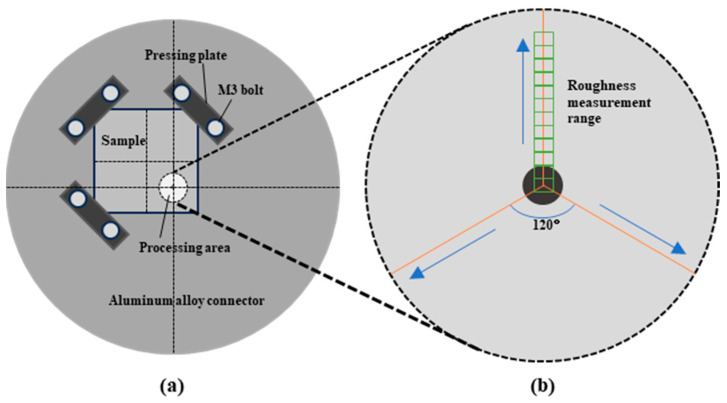
(**a**) Schematic diagram of the connecting plate made of the aluminum alloy specimen; (**b**) surface roughness measurement method.

**Figure 3:**
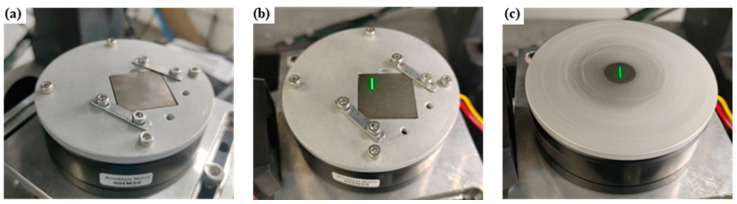
Schematic diagram of rotary laser ablation: (**a**) install the specimen, (**b**) move the laser to the appropriate position, (**c**) schematic diagram of the laser processing.

The aluminum alloy connector was designed to fix the specimen onto the hollow brushless motor. The design of the aluminum alloy connecting plate is shown in Figure 2a and the physical object is shown in Figure 3a. The design of the sinking installation test piece means that it can be safely fixed to the test piece and one test piece can be used to carry out four sets of tests.

**Figure 4:**
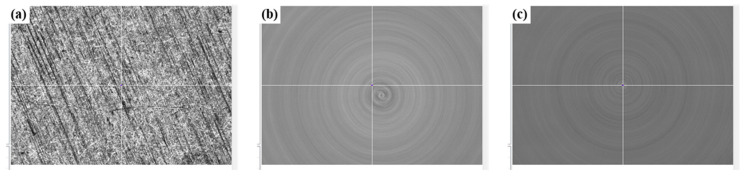
CCD images during the process of finding the machining area: (**a**) non-rotating surface, (**b**) rotating surface, (**c**) aligned surface.

Before laser processing, the machine tools were controlled near the predetermined ablation position, the focal length was adjusted, and the CCD was turned on. The surface of the titanium alloy can be observed as shown in Figure 4a. Turn on the brushless motor and adjust it to the lowest rotational speed to avoid motor heating caused by long-term high-speed operation. The image of the rotating sample displayed by the CCD is shown in Figure 4b. Through the high-precision control of the incremental coordinates of the machine tools, the rotation center is aligned with the cross-center of the CCD image auxiliary line, as shown in Figure 4c.

The TC4 titanium alloy was used as the test material. The chemical composition is shown in Table 1. Each parameter in the test was processed once. After the test piece was installed, the laser power was measured using a power meter (OPHIR), the hollow brushless motor was turned on, the speed was adjusted, the laser was turned on after the speed was stabilized, and the ablation timing was stopped after 8 s. After the test, a circular processing area with a diameter of 5.3 mm was generated, covering the processing area of 22.05 mm^2^. After completing a set of tests, the position of the specimen was adjusted and the abovementioned steps were repeated for the next test.

After the experiment, the surface morphologies after ablation were observed by a confocal microscope (VK-X200K, Keyence, Osaka, Japan) and the surface roughness of each region was measured at a maginification of 1000 times. The microstructure changes before and after the test were observed by a thermal field emission scanning electron microscope (Verios G4 UC, Thermo Scientific, Waltham, MA, USA), and the element type and content on the surface of the specimen were detected using an accessory X-ray energy spectrometer (Oxford X-Max 50, Oxford, UK). An X-ray powder diffractometer (D8 Advance, Bruker, Lücken, Germany) was used to qualitatively and quantitatively analyze the surface elements of the material before and after the test. The XRD target used was a copper target and the X-ray wavelength generated by the equipment was 1.5406 Å.

A total of three radii were selected, at an angle of 120° in the circumferential direction of the processing area. The surface morphology and data were measured by confocal microscopy every 200 μm along these radii. In terms of the three radii, the surface roughness of the area with the same distance from the ablation center was averaged to ensure the reliability of the data, as shown in Figure 2b. The surface topography and surface roughness were measured every 200 μm along the radius of each processing area, and the distance between the measurement area and the ablation center was recorded, which is defined as the measurement radius ($R_{m}$). The radius of each processing area was measured for 13 groups and the measurement radius ranged from 0 to 2400 μm, as shown in Figure 2b. The surface roughness was measured at a distance along each radius, and the surface roughness corresponding to the measurement radius was obtained by averaging the surface roughness of the same measurement radius. Then, the changes in the surface morphologies of the corresponding position were observed.

The conversion relationship between the angular velocity ω and the rotational speed n is:

(1) ω=2πn

The transformation relationship between the measurement radius $R_{m}$ and the scanning speed ν is:

(2) $$v=\omega R_{m}$$

The relationship between the scanning speed ν, the motor speed n, and the measurement radius $R_{m}$ is inferred. It can be seen that the scanning speed is proportional to the measurement radius:

(3) $$v=2\pi nR_{m}$$

The conversion relationship between the scanning speed ν, the spot overlap rate Overlap, the laser repetition frequency f, and the spot width D is as follows:

(4) $$Overlap=(1-\frac{v}{D\times f})$$

This study consisted of two parts, namely the variable rotational speed test and the variable power test, which were tested on sample 1 and sample 2, respectively. The first part of the variable rotational speed test parameters is shown in Table 2. The adjusted rotational speed n involved a range of adjustment from 0 rpm to 3000 rpm and the numerical adjust spacing was 600 rpm, to explore the influence of the rotational speed on the rotating ablation of the line spot. The second part of the variable power test parameters is shown in Table 3. The laser power was adjusted from 11 W to 14 W and the numerical adjust spacing was 1 W, to explore the influence of laser power on the ablation of the rotational line spot. During the test, the repetition frequency was 10 kHz and the ablation time was 8 s.

## 3. Results and Discussion

### 3.1. Surface Roughness and Surface Morphologies

#### 3.1.1. Effects of Rotational Speed on the Surface Roughness and Surface Morphologies

**Figure 5:**
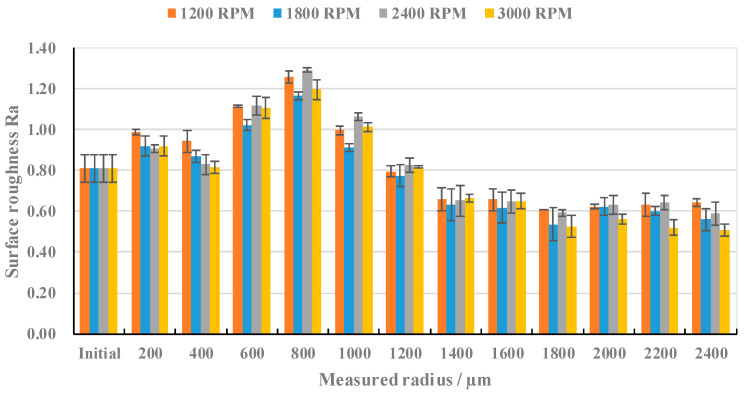
The variation trend in the surface roughness according to the $R_{m}$, at different rotational speeds.

The rotational speed was adjusted according to the experimental parameters described in Table 2, and the change trend in the surface roughness of Sample 1 with the corresponding measurement radius was recorded, as shown in Figure 5. Under the conditions of a laser power of 14 W and an ablation time of 8 s, the change trend in the surface roughness with the corresponding measurement radius at different rotational speeds was approximately the same, showing a trend of rising first, then falling, and then rising. The molten material was produced by a nanosecond laser on the surface of the material, and high-speed rotation will have an impact on the distribution of molten material, which will induce the effect of inertia and centrifugal force; the reconsolidation of molten material has a greater impact on the formation of the surface morphology. The distribution of the molten pool on the surface makes no significant difference in terms of the curve of the surface roughness, with the corresponding measurement radius, at different rotational speeds. When the measurement radius is 200 μm, the surface roughness reaches the maximum value for the first time. When the measurement radius is 800 μm, the surface roughness reaches its peak. After the measurement radius reaches 800 μm, the surface roughness decreases slowly with the increase in the measurement radius and tends to be stable after reaching 1800 μm.

**Figure 6:**
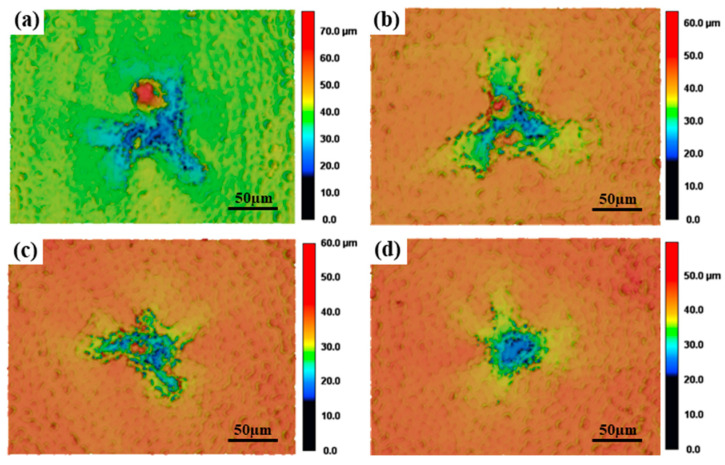
Surface morphologies of the rotating center at different rotational speeds: (**a**) 1200 RPM, (**b**) 1800 RPM, (**c**) 2400 RPM, (**d**) 3000 RPM.

As shown in Figure 6, a pit appears in the ablation center and the height of the pit has large fluctuations, with a conical bulge and a shallow pit, like a fan blade, around the pit. As the rotational speed increases, the shallow pit around the pits become shallow and the height of the bulge decreases, until it disappears.

**Figure 7:**
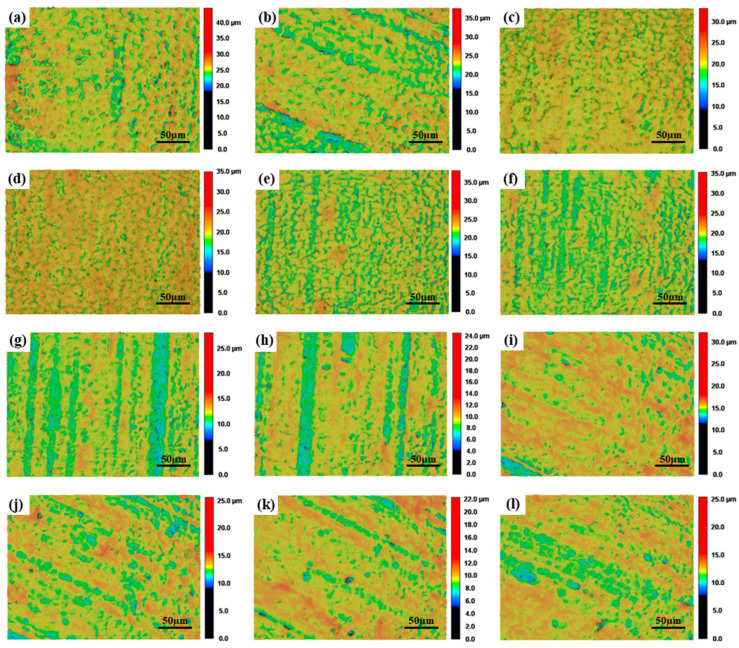
Color height maps of the surface morphologies with the variation in the $R_{m}$ at the magnification of 1000 times when the rotational speed is 3000 RPM: (**a**) 200 µm, (**b**) 400 µm, (**c**) 600 µm, (**d**) 800 µm, (**e**) 1000 µm, (**f**) 1200 µm, (**g**) 1400 µm, (**h**) 1600 µm, (**i**) 1800 µm, (**j**) 2000 µm, (**k**) 2200 µm, (**l**) 2400 µm.

As shown in Figure 7a,b, in the $R_{m}$ range of 200 μm to 600 μm, the laser scanning speed on the surface of the material is small, the laser residence time [16] on the surface of the specimen is long, surface over melt occurs, and the melting depth of the SOM region is large. When the laser energy density is too large, The roughness of the machined surface will increase compared to the original surface roughness [18]. Under the laser confocal microscope with a maginification of 1000 times, a ripple pattern was observed on the polished surface of the specimen after solidification. A large number of ripples result in the surface roughness reaching the maximum value, when the measurement radius is 200 μm. With the increase in the measurement radius, the laser scanning speed increases, the surface ripple of the specimen decreases gradually, the surface roughness decreases slightly, and microcracks appear and increase gradually with the increase in the measurement radius [19]. The appearance of microcracks gradually increases the surface roughness and the peak is reached when the measurement radius is 800 μm.

As shown in Figure 7d–i, in the $R_{m}$ range from 800 μm to 1800 μm, the surface roughness gradually decreases, and the surface of the specimen gradually changes from surface over melt to surface shallow melt (SSM) [26]. Surface shallow melt, manifested in the material surface-melting part of the material, experiences its own gravity and, the role of surface tension, causes the flow and fill of the material surface’s depressions and cracks; liquid melt cooling and solidification of the surface of the curvature of the various parts of the surface tends to be the same, meaning that the new surface’s flatness increases dramatically in order to achieve the purpose of reducing the surface roughness. Surface over melt is manifested by too high a laser power or too low a scanning speed, which will lead to material surface residence time of the laser being too long and the formation of a molten pool, and thus the phenomenon of surface over-melting occurs; the roughness of the surface of processed material will be reflected in the periodic distribution of the structure, such a structure may make the original surface roughness increase instead of decrease. After the $R_{m}$ reaches 1800 μm, the surface roughness gradually becomes stable. When the $R_{m}$ exceeds 1800 μm, the centrifugal force generated by the rotation of the specimen has a certain influence on the surface roughness and the trend in the roughness variation tends to be gentle, making it difficult to further reduce. When the line spot length and the processing area is large enough, the surface roughness will gradually tend towards the original surface roughness.

#### 3.1.2. Effects of Laser Power on Surface Roughness and Surface Morphologies

**Figure 8:**
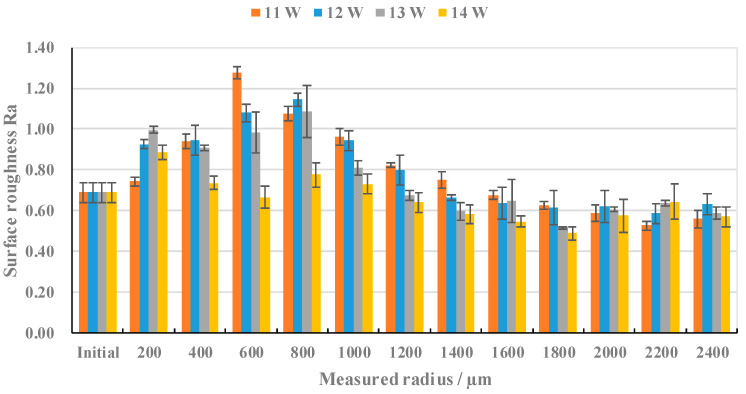
The variation trend in surface roughness according to the $R_{m},$ under different laser powers.

**Figure 9:**
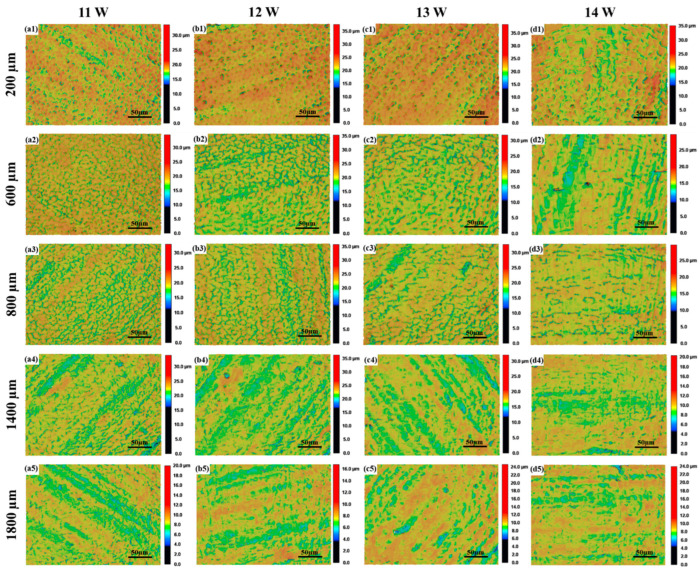
Color height diagrams of the surface morphologies with different $R_{m}$, under different laser powers: (**a1**) *P* = 11 W, *R_m_* = 200 µm, (**a2**) *P* = 11 W, *R_m_* = 600 µm, (**a3**) *P* = 11 W, *R_m_* = 800 µm, (**a4**) *P* = 11 W, *R_m_* = 1400 µm, (**a5**) *P* = 11 W, *R_m_* = 1800 µm, (**b1**) *P* = 12 W, *R_m_* = 200 µm, (**b2**) *P* = 12 W, *R_m_* = 600 µm, (**b3**) *P* = 12 W, *R_m_* = 800 µm, (**b4**) *P* = 12 W, *R_m_* = 1400 µm, (**b5**) *P* = 12 W, *R_m_* = 1800 µm, (**c1**) *P* = 13 W, *R_m_* = 200 µm, (**c2**) *P* = 13 W, *R_m_* = 600 µm, (**c3**) *P* = 13 W, *R_m_* = 800 µm, (**c4**) *P* = 13 W, *R_m_* = 1400 µm, (**c5**) *P* = 13 W, *R_m_* = 1800 µm, (**d1**) *P* = 14 W, *R_m_* = 200 µm, (**d2**) *P* = 14 W, *R_m_* = 600 µm, (**d3**) *P* = 14 W, *R_m_* = 800 µm, (**d4**) *P* = 14 W, *R_m_* = 1400 µm, (**d5**) *P* = 14 W, *R_m_* = 1800 µm.

According to the experimental parameters described in Table 2, the laser power was adjusted and the change trend in the surface roughness of sample 2 with the corresponding measurement radius was recorded, as shown in Figure 8. Under the microscope observation of the same magnitude, the $R_{m}$ ranged from 0 μm to 2400 μm and the surface morphology variations in terms of the different laser powers were similar to the variable speed test. They are all central pits to ripples and, then, they go from ripples to reticulated pits. Then, the reticulated pits gradually decrease and the surface gradually becomes smooth, as shown in Figure 9.

Under the influence of a laser power of 13 W and 14 W, the surface roughness of the processing area experienced the same change trend according to the measurement radius, which first rises to the maximum, and then decreases slightly, and then rises again. The surface roughness reaches its peak at an $R_{m}$ of 800 μm, and then decreases slowly, and tends to be stable. When the $R_{m}$ is 200 μm, the scanning speed is low, the laser stays on the surface of the material for a long time, and the surface of the specimen is in a state of surface over melt. Due to the long existence time of the molten pool, the molten pool oscillates under the combined action of the Marangoni effect, surface tension, and gravity [27]. Due to the influence of the Marangoni effect, the surface roughness increases and reaches the first maximum value. In the $R_{m}$ range from 400 μm to 1800 μm, at the same $R_{m}$, with the increase in laser power, the surface roughness decreases gradually. This is due to the melting of the surface metal caused by the interaction between the laser and the surface of the material. The increase in laser power increases the melting degree of the surface of the specimen, and the molten metal fills the peaks and valleys on the surface of the specimen, and the surface roughness decreases.

Under the action of 11 W and 12 W laser power, the surface roughness of the machined area with the corresponding measurement radius has a similar trend, both of them first increase to reach a great value, and then fall, and then experience dynamic stabilization. Under the influence of 11 W laser power, the surface roughness reaches its peak when the $R_{m}$ is 600 μm. Since the low laser power cannot provide sufficient laser energy, the measurement radius corresponding to the surface roughness peak of the microcrack area is reduced, and the peak is reached at 600 μm.

After the $R_{m}$ reaches 1800 μm, the surface roughness increases slightly with the increase in the measurement radius, and finally tends toward the original surface roughness. This is because the scanning speed of the corresponding area is large, the laser residence time on the surface of the material is short, and the laser energy makes the surface melting degree low, or it is not enough to make the surface of the material melt.

**Figure 10:**
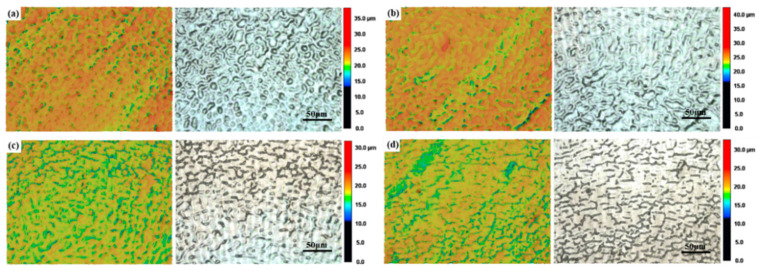
Surface topography and line spot characteristic change trends at different measurement radii under a laser power of 13 W: color height map (left) and black and white texture map (right) for (**a**) 200 µm, (**b**) 400 µm, (**c**) 600 µm, (**d**) 800 µm.

During the process of laser polishing, the molten metal is driven by the thermocapillary force to move to the center of the molten pool and produce linear protrusions. The lower $R_{m}$ corresponds to the lower scanning speed. The interaction between the laser energy and the material surface is sufficient, which will cause obvious protrusions and produce linear traces [25]. As shown in Figure 10, there are obvious linear features on the surface of the specimen under the action of the 13 W laser power, and with the increase in the measurement radius, the linear traces gradually decrease, until they disappear.

### 3.2. Micro-Morphologies and Microstructure Analysis

#### 3.2.1. Microscopic Morphologies

**Figure 11:**
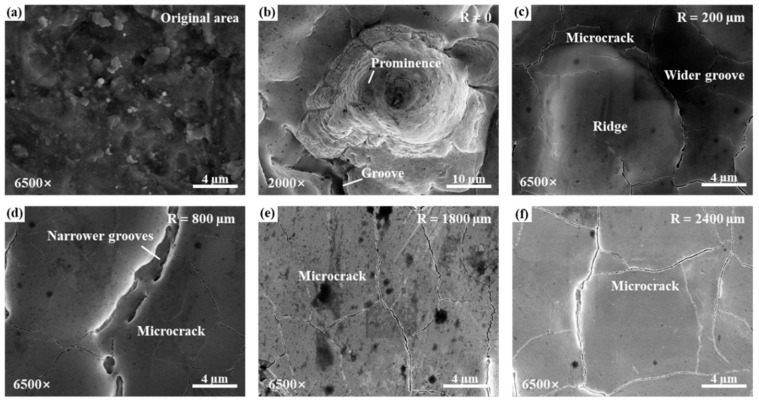
The uncleaned morphologies under a scanning electron microscope under a laser power of 13 W: (**a**) original surface, (**b**) surface morphology of ablation center, (**c**) R = 200 μm, (**d**) R = 800 μm, (**e**) R = 1800 μm, (**f**) R = 2400 μm.

**Figure 12:**
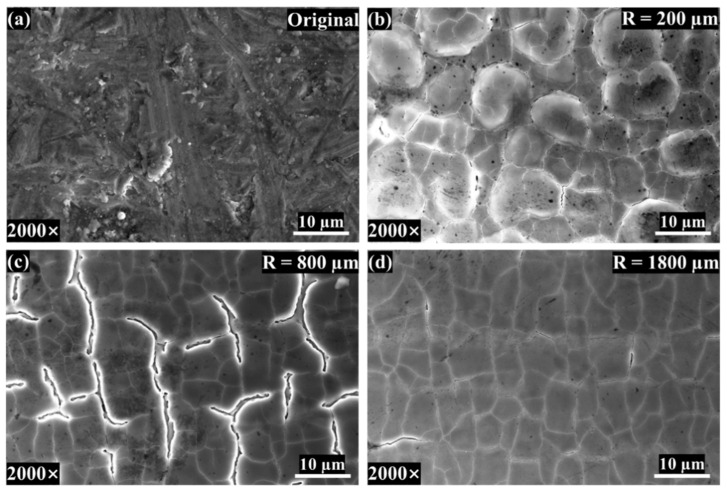
The surface morphologies of different measurement radii under a laser power of 13 W: (**a**) original surface, (**b**) 800 µm, (**c**) 800 µm, (**d**) 1800 µm.

**Figure 13:**
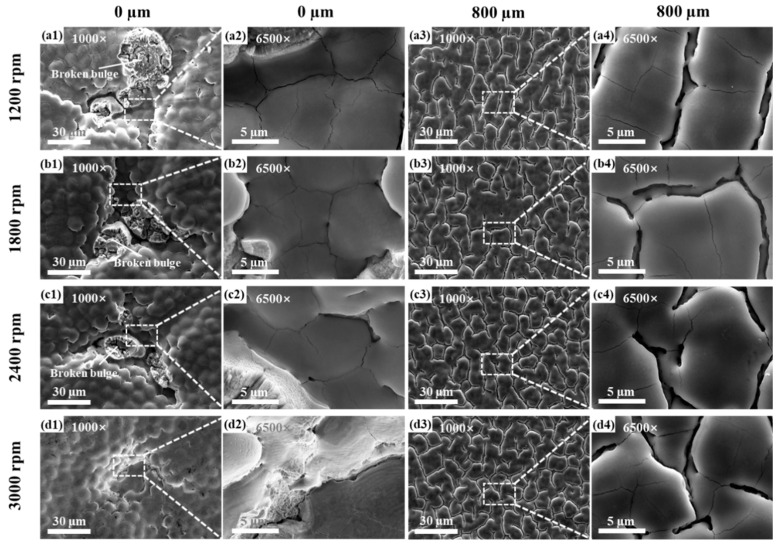
The micro-morphologies of the ablated surface at different multiples of the rotational center after cleaning and a measurement radius of 800 μm corresponding to different rotational speeds under a laser power of 14 W: (**a1**–**a4**) 1200 rpm, (**b1**–**b4**) 1800 rpm, (**c1**–**c4**) 2400 rpm, (**d1**–**d4**) 3000 rpm.

In order to further analyze the variation in the micro-surface morphologies of the rotating laser ablation under different measurement radii, the laser ablation surface under different multiples was observed by a scanning electron microscope. The original micro-morphology of the sample and the micro-morphology of different measurement radii under a 13 W laser and 2000 RPM test conditions are shown in Figure 11, Figure 12 and Figure 13. The original surface was rough and uneven. After the rotational center was ablated by a 13 W laser, bumps and grooves were generated, as shown in Figure 11b and Figure 12. There were microcracks at the bottom of the grooves. In Section 3.1, the cause of the central bulge was related to the rotational speed. Figure 11c shows the surface morphology when the $R_{m}$ is 200 μm, with uplift characteristics, wide grooves, and densely distributed microcracks. As the measurement radius increases, the distribution of the microcracks gradually becomes sparse, but the width of the microcracks increases slightly, as shown in Figure 11c–f. When the $R_{m}$ is 800 μm, the uplift in the surface distribution is not obvious, and the groove becomes narrow and deep, as shown in Figure 11d. The slow scanning speed increases the residence time of the laser on the surface of the material, resulting in surface over melt (SOM), while the thermocapillary force tends to drive the melt to the center of the molten pool [28], resulting in bulges and wider grooves. The active oxygen element in the air also promotes the thermocapillary force to drive the melt to the center of the molten pool [29].

The micro-morphology variations for different $R_{m}$ after 13 W laser irradiation are shown in Figure 12. When the measurement radius is 200 μm, the surface ripples observed under the confocal microscope show a relatively uniform distribution of approximately circular bulges at the micro level. At this time, the scanning speed is low, the laser and the material interaction time is long, and the molten pool exists for a long time. The existence of the Marangoni effect causes the molten pool to oscillate, and the surface roughness is large. At this time, the Marangoni effect acts in conjunction with gravity and surface tension, and the cooling of the melt pool occurs as re-solidification occurs, producing a bulge and surface over-melt phenomenon [27]. When the $R_{m}$ is 800 μm, the surface is subjected to capillary force and thermocapillary action, and the surface roughness reaches its peak. When the $R_{m}$ is 1800 μm, the molten liquid metal fills in the peaks and valleys on the specimen’s surface due to gravity, the capillary force, and the thermocapillary force [30]. After the $R_{m}$ reaches 1800 μm, with the increase in the measurement radius, the laser energy absorbed by the material is not enough to make the molten metal flow [25], and the variation in the surface roughness tends to be stable.

Under the influence of the 14 W laser, the surface morphologies after cleaning at different speeds are shown in Figure 13. After cleaning, the ablation’s central bulge was damaged and fell off, revealing an internal stripe-like structure. As the rotational speed increases, the central bulge becomes smaller, and the bulge is not obvious or even disappears at 3000 rpm, as shown in Figure 13(a1–d1). Microcracks are distributed at the bottom and bulge in the central ablation groove. The micro-morphology with an $R_{m}$ of 800 μm at different rotational speeds resulted in a surface that was densely distributed with grooves and microcracks, and the corresponding surface roughness was similar, indicating that the surface morphology and roughness were not significantly affected by the rotational speed.

#### 3.2.2. Element Distributions

**Figure 14:**
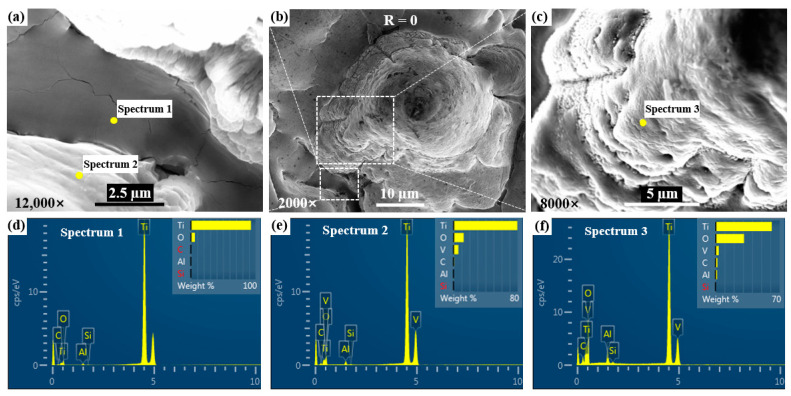
The proportion of point scanning elements on the surface of the uncleaned ablation center under a laser power of 13 W: (**a**) the surface morphology of the central groove, (**b**) the surface morphology of the central surface, (**c**) the surface morphology of the central bulge, (**d**) the energy spectrum 1 at the bottom of the groove, (**e**) the energy spectrum 2 at the side wall of the groove, (**f**) the energy spectrum 3 at the top of the bulge.

The surface of the machined specimen after being subject to a laser power of 13 W was analyzed by scanning element analysis using an energy dispersive spectrometer and a scanning electron microscope. The surface morphologies and point scanning energy spectrum of the central groove under a magnification of 12,000 times and the uncleaned ablation central bulge under a magnification of 8000 times are shown in Figure 14. It can be seen that the content of the O element increases gradually from the bottom of the groove to the side wall of the groove, and then to the central bulge. This is due to the fact that the Ti element in the bottom of the groove has less contact with the O element in the air, and the trace oxidation reaction occurs. On the contrary, the Ti element in the bulge is more in contact with the oxygen in the air, and the oxidation reaction is more intense, and the O element content in the point scanning energy spectrum is higher. The TC4 titanium alloy contains trace amounts of V and Al elements. After an oxidation reaction at a high temperature, V_2_O_5_ and Al_2_O_3_ are produced and precipitated on the surface, resulting in less V and Al content at the bottom of the groove.

**Figure 15:**
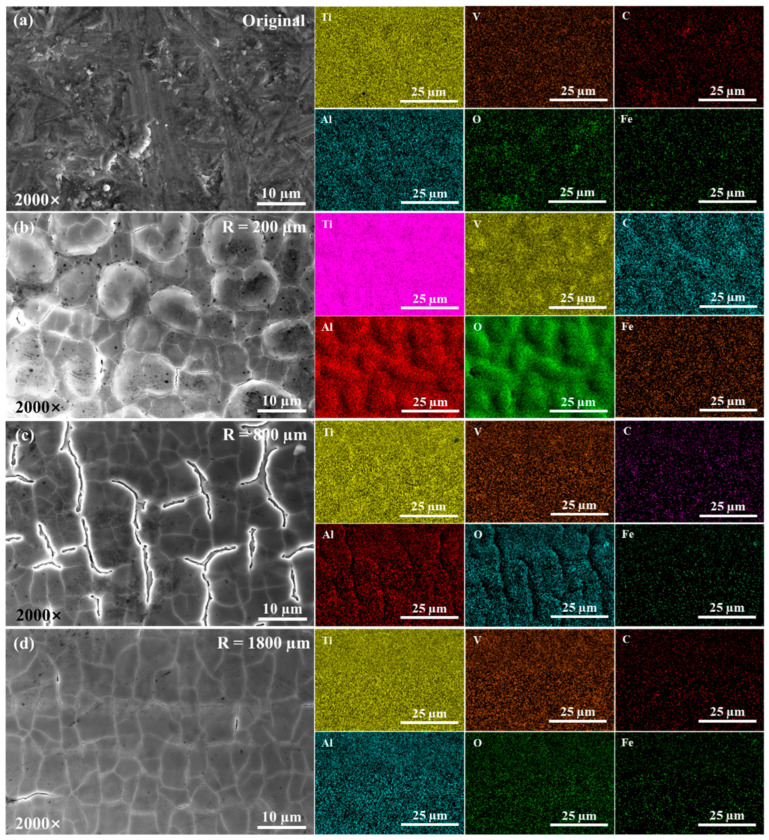
The surface scanning element distributions of the uncleaned specimen surface under a laser power of 13 W: (**a**) original surface, (**b**) R = 200 μm, (**c**) R = 800 μm, (**d**) R = 1800 μm.

The surface morphologies and surface scanning element distribution under different $R_{m}$ are shown in Figure 15. The main element in the original surface was Ti, containing a small amount of V and Al, trace amounts of Fe, C, O, and other impurities. When $R_{m}$ is 200 μm, the O content increases sharply. When $R_{m}$ is 800 μm, the O content is lower than that when $R_{m}$ is 200 μm. When $R_{m}$ is 200 μm and 800 μm, the distribution of the Al element is similar to that of the O element. At this time, in addition to the oxidation of the Ti element to form TiO_2_, the Al and V elements are also oxidized, and the oxidized Al_2_O_3_ and V oxides are formed, which are precipitated and distributed on the surface rises. The change in the surface roughness indicates that surface over melt has occurred in this area and the transition from surface over melt to surface shallow melt gradually occurs after the measurement radius exceeds 800 µm. With the increase in the measurement radius, when the measurement radius is 1800 μm, the element distribution is uniform and the content of the O element is similar to that of the original surface. At this time, the surface is in the shallow melting state and the surface is not obviously oxidized. The molten metal fills the peaks and valleys. However, due to the high scanning speed and the centrifugal force generated during the rotation process, the surface of the specimen is continuously smooth, but there are microcracks after rapid cooling and solidification.

#### 3.2.3. Analysis of XRD Pattern

**Figure 16:**
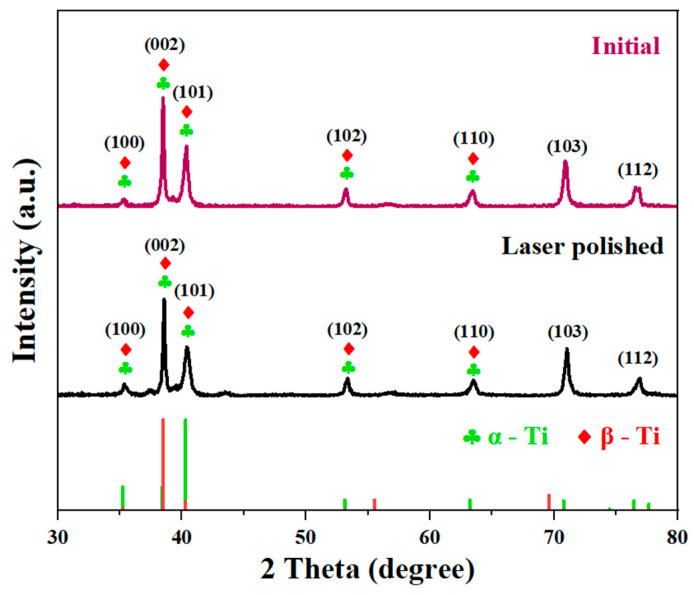
X-ray diffraction patterns for the initial surface and laser-polished surface.

The surface of the sample affected by the 13 W laser was compared with the results of the XRD detection on the initial surface, to further study the variation in the rotational laser ablation surface. As shown in Figure 16, the TC4 titanium alloy is a dual-phase titanium alloy. The α phase with a hexagonal close-packed structure and the β phase with a body-centered cubic structure constitute the basic phases of the TC4 titanium alloy. The XRD pattern is characterized by (100), (002), (101), (102), (110), (103), and (112), a total of seven main diffraction peaks. After rotating ablation, the (101) diffraction peak decreased slightly, and the other diffraction peaks did not change significantly. The overlap in the diffraction peaks make it difficult to distinguish between the α phase and the β phase. However, from the analysis of the fitting results in the full diagram of the peaks, (002) is the highest diffraction peak in the initial surface of the specimen and the β phase has a higher proportion. The diffraction peak in the α phase is (101), and the diffraction peak in the rotational ablation surface (101) decreases slightly, indicating that a small part of the α phase on the surface of the specimen is transformed into the β phase after rotating laser ablation. Available TC4 polishing, studies have found that [31] when the surface temperature of the TC4 specimen increases to the phase transition threshold, the transition from the α phase to the β phase occurs. During cooling, according to the different cooling rates, the β phase will decompose into secondary α or martensite α’. The spot overlap rate of the rotational ablation test in this study decreases with the increase in the measurement radius. The residence time of the spot on the surface of the specimen is small and the energy absorbed by the specimen is small. Only a small part of the ablation area in the center can reach the phase transition threshold. Unlike previous studies, a small portion of the α phase produced by the ablation center decomposes into the β phase during cooling, while most of it remains in the α phase, resulting in a decrease in the (101) diffraction peak. The reason for the decrease in the (101) diffraction peak may also be due to the reorientation of the grains inside the polycrystalline sample caused by laser polishing, and the relevant research conclusions need to be further explored.

## 4. Conclusions

In this paper, a new laser processing method for the TC4 titanium alloy was investigated, and the interaction mechanism between a rotating nanosecond modulated laser and the TC4 titanium alloy was analyzed. The variation in the surface roughness and morphologies of laser ablation, along with the laser power and rotational speed, under various measurement radii, were studied. The main conclusions are as follows:

(1) Under the various rotational speeds and powers, the measurement radii ranges from 0 μm to 2400 μm and the surface morphologies present a similar variation trend. The central part of the ablation will show a concave phenomenon, with densely distributed micro-uplifts and then the uplifts gradually flatten out. Firstly, dense grooves appear and, eventually, the grooves disappear, leaving only microcracks. In the variable speed experiments, the central bulge and the blade-like pit gradually decrease and disappear with the increase in the rotational speed, and the edge shape of the central pit gradually tends to be circular;

(2) The surface roughness shows a certain rule with the variation in the measurement radius. In the variable power experiments, the surface roughness reaches its peak when the measurement radius is 800 μm, under a laser power of 12 W, 13 W, and 14 W. When the laser power is 11 W, the measurement radius of the surface roughness reaches its peak value at 600 μm. The lower laser power may advance the transition zone between surface over-melting and shallow surface melting. There are obvious linear traces in the surface corresponding to the small measurement radius. As the measurement radius increases, the spot overlap rate decreases, and the surface of the surface transitions from surface over melt to surface shallow melt, when the laser energy characteristic that acts on the surface materials varies;

(3) After ablation, an Al-rich oxide is produced at the center of the surface, the O content at the bottom of the ablation center pit is low, and the O content on the convex surface is high. When the measurement radius is 800 μm, the O content increases further, and surface over-melting occurs. With the increase in the measurement radius, there is no significant difference between the O content and the initial surface at 2400 μm;

(4) Due to the different measurement radii of rotational laser ablation, the laser energy irradiated on the unit area of the specimen’s surface per unit of time is different. At the ablation center, due to the large amount of laser energy absorbed by the material, the temperature reaches the phase transition threshold and the phase transition from the α phase to the β phase occurs.

Compared with the traditional laser processing spot, the rotary linear spot covers a larger processing area of 22.05 mm^2^, which has significant application potential in the field of high-efficiency and high-quality laser processing. This article mainly conducts basic process research and speculates on the possible reasons for such occurrence. The influence of centrifugal force will be conducted in further in-depth research and discussion in the future. This research work can be used as a research basis for rotary modulation laser surface processing and has significance for guiding the research and development of high-quality and high-efficiency laser processing technology.

## Figures and Tables

**Table 1:** The mass ratio of the chemical composition of the TC4 titanium alloy.

Material	Al	V	Fe	Ti
TC4	5.5–6.8	3.5–4.5	<0.30	Balance

**Table 2:** Parameters of the rotational line spot, variable rotational speed, ablation experiment involving the TC4 titanium alloy.

Experimental Parameters	Parameter Value
Laser repetition frequency *f*/(kHz)	10
Laser power *P*/(W)	14
Rotational speed *n*/(r/min)	0, 1200, 1800, 2400, 3000
Ablation time/(s) Beam spot size Material sample size	8 14 μm × 5.3 mm 25 mm × 25 mm × 1 mm

**Table 3:** Parameters of the rotational line spot, variable laser power, ablation experiment involving the TC4 titanium alloy.

Experimental Parameters	Parameter Value
Laser repetition frequency *f*/(kHz)	10
Laser power *P*/(W)	11, 12, 13, 14
Rotational speed *n*/(r/min)	2000
Ablation time/(s)	8
Beam spot size	14 μm × 5.3 mm
Material sample size	25 mm × 25 mm × 1 mm

## Data Availability

The original contributions presented in the study are included in the article, further inquiries can be directed to the corresponding author.
